# Gap Shape Classification using Landscape Indices and Multivariate Statistics

**DOI:** 10.1038/srep38217

**Published:** 2016-11-30

**Authors:** Chih-Da Wu, Chi-Chuan Cheng, Che-Chang Chang, Chinsu Lin, Kun-Cheng Chang, Yung-Chung Chuang

**Affiliations:** 1 Department of Forestry and Natural Resources, College of Agriculture, National Chiayi University, Chiayi, 60004, Taiwan; 2 The Department and Graduate Institute of Landscape Architecture, Chinese Culture University, Taipei, 11114, Taiwan; 3 Technical Service Division, Taiwan Forestry Research Institute, Taipei, 10066, Taiwan; 4 Department of the Urban Planning and Spatial Information, Feng Chia University, Taichung, 407, Taiwan

## Abstract

This study proposed a novel methodology to classify the shape of gaps using landscape indices and multivariate statistics. Patch-level indices were used to collect the qualified shape and spatial configuration characteristics for canopy gaps in the Lienhuachih Experimental Forest in Taiwan in 1998 and 2002. Non-hierarchical cluster analysis was used to assess the optimal number of gap clusters and canonical discriminant analysis was used to generate the discriminant functions for canopy gap classification. The gaps for the two periods were optimally classified into three categories. In general, gap type 1 had a more complex shape, gap type 2 was more elongated and gap type 3 had the largest gaps that were more regular in shape. The results were evaluated using Wilks’ lambda as satisfactory (p < 0.001). The agreement rate of confusion matrices exceeded 96%. Differences in gap characteristics between the classified gap types that were determined using a one-way ANOVA showed a statistical significance in all patch indices (p = 0.00), except for the Euclidean nearest neighbor distance (ENN) in 2002. Taken together, these results demonstrated the feasibility and applicability of the proposed methodology to classify the shape of a gap.

Canopy gap characteristics are significantly related to forest composition and life dynamics. Forest communities that are disturbed by various factors exhibit canopy gaps during the succession stage^1^. Most natural, small-scale disturbances are so well integrated into community dynamics that they are considered keystone processes for maintaining the health or integrity of ecosystems. Canopy gaps and the agents that cause gaps influence many different forest resource values^2^. Canopy gaps are thought to be important structural components that influence the forest ecosystem’s environment, functions and processes, such as the mortality and growth of surrounding trees^3^, the duration of sunshine and soil moisture in forest^3^, plant competition and regeneration^4^, the success rate of mature tree seedlings^5^, and the location of coarse woody debris^6^. Gaps in the forest canopy that are created by disturbance agents are effective indicators of the health and stability of a large-area forest^2^. Canopy gap and individual tree growth records also provide important information that allows changes in the forest environment to be predicted^7^,^8^,^9^. There are strong links between forest ecology and canopy gaps^10^,^11^,^12^.

There are many factors that generate canopy gaps: single plant death, windstorms, lightning, forest fires, landslides, or human disturbance. Canopy gaps that are generated by various disturbances differ in size, shape and pattern, which determines local variation in their function in forest ecosystem services, micro-climate regulation, species diversity and forest succession^3^,^13^,^14^,^15^,^16^. Gaps of different shapes and pattern characteristics are also indicators of the forest succession stage and the constitution of the vegetation. However the huge variation in gap shape results in difficulty in determining the association between canopy gap characteristics and forest ecosystems. By grouping canopy gaps with similar characteristics together, the complexity of determining forest ecosystem by gap shapes could be reduced. No studies have classified gap shape to determine its effect on related issues, such as the interpretation of the type of forest vegetation or forest composition analysis.

Landscape ecology is largely founded on the notion that environmental patterns strongly influence ecological processes^17^. A disruption in landscape patterns can therefore compromise functional integrity by interfering with critical ecological processes that are necessary for the persistence of a population and the maintenance of the biodiversity and health of an ecosystem^18^. Therefore, much emphasis is placed on developing methods to quantify landscape patterns, which is considered a prerequisite to the study of pattern-process relationships^19^. This has resulted in the development of literally hundreds of indices for landscape patterns. This progress has been facilitated by recent advances in computer processing and geographic information (GIS) technologies^19^. Currently, much effort is being devoted to using these quantified landscape indices to analyze landscape change and to study the effect of disturbance on the landscape pattern, such as the effects of forestry cutting and forestry roads on the fragmentation of the landscape’s structure^20^,^21^. Since landscape indices are functional indicators that represent the shape, attributes, distribution and composition of individual patches, information about the characteristics of the shape of gaps can be obtained by calculating landscape indices.

This study proposes an innovated methodology to classify canopy gaps according to their shape characteristics. The results could allow forestry scientists and ecology professionals to determine the complicated composition and distribution of forest vegetation using information that is derived from the proposed methodology.

## Results

### Gaps for the two periods

The numbers of gaps and the gap percentages over the entire study area in 1998 and 2002 are shown in Table S1. Gap counts increased from 218 to 301. However, the gap area decreased slightly from 45816 m^2^ to 45397 m^2^, which showed that despite a significant increase in the number of gaps, the total area of gaps over the entire landscape remained almost unchanged. Therefore, it could be inferred that small gaps had been increasing in the Lienhuachih natural protected forestry area. Table S2 shows the unpaired t-test for gap shape characteristics for the two periods. Five landscape indices, including PERIM, GYRATE, PARA, FRAC and CONTIG, showed a statistical difference (p < 0.05). The gaps in 2002 were also smaller in area and more irregular in shape and show greater contiguity, or connectedness, than those in 1998. Table S3 shows the correlation coefficients for the ten patch indices in 1998. AREA, PARA, FRAC, CONTIG, PROX and ENN were not collinear and these were initially used for the first attempt at a non-hierarchical cluster analysis afterward.

### Gap classification

The results of the one-way ANOVA during the cluster procedure showed that PROX did not achieve statistical significance (p > 0.05) and was not a functional variable for gap type classification. Therefore, PROX was removed and the cluster procedures rebuilt. The calculated CCC values increased from 1.09 to 7.05 and then dropped to 2.84 when the number of clusters increased from two to four, which implied that the optimal number of gap clusters for 1998 was three.

A canonical discriminant analysis generated the discriminant functions and classified the gaps for 1998 into three categories. All five patch indices were used (p < 0.01) for the discriminant functions. Table 1 shows a comparison of gap types for 1998, using a one-way ANOVA. The differences between the three indices were statistically significant (p < 0.01), which showed that the three gap types classified had different characteristics, in terms of shape and pattern. Table 2 shows the standardized canonical discriminant function coefficients for 1998. According to the coefficient estimates, CONTIG and PARA were the respective dominant variables for Functions 1 and 2. Table S4 lists the model Wilks’ lambda for the developed functions. The estimates for Wilks’ lambda for the two functions were 0.15 and 0.94 (p < 0.01 for both cases), which demonstrated the ability of the developed functions to distinguish.

Table S5 shows the confusion matrices for self-classification and cross-validation. Agreement rate for the different approaches was consistently greater than 96%. These evaluation results confirmed the performance of the established discriminant functions. The differences in the characteristics for the three gap types for 2002 (Table 3) were also examined and all patch indices showed a statistical significance (p = 0.00) except for ENN (p = 0.83). In general, these results showed that the classified gap categories had distinctive features, in terms of size, shape and spatial configuration.

**Figure 1:**
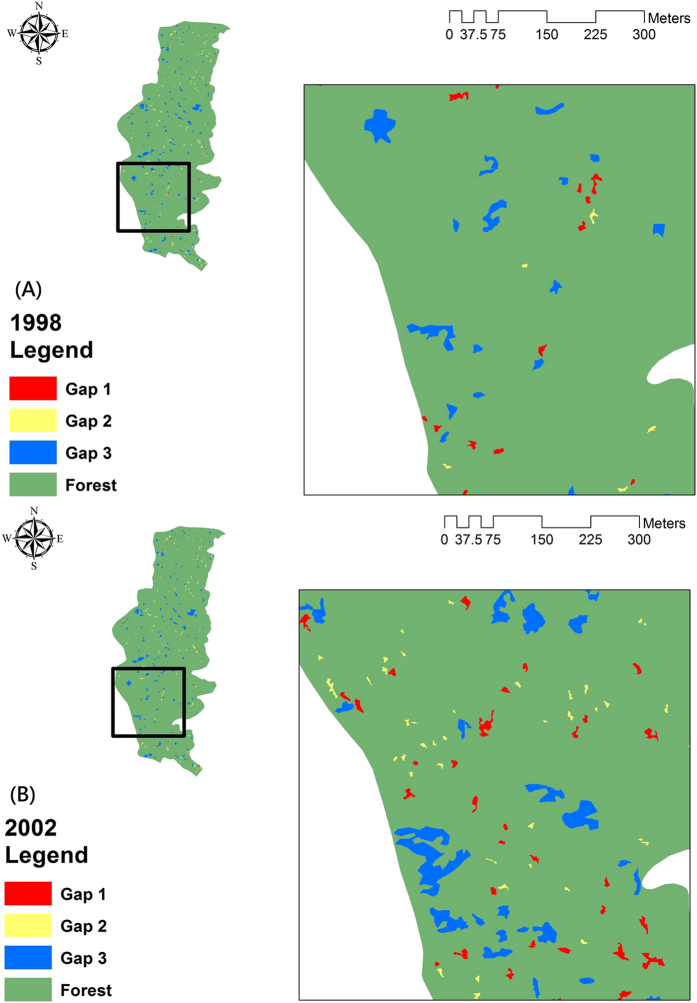
The classified gap maps of (**A**) 1998 and (**B**) 2002. Esri ArcGIS 10.2 was used to create this figure (http://www.esri.com/software/arcgis).

Figure 1 shows the classified gap maps for (A) 1998 and (B) 2002. As shown, there were three gap types: 1, 2, and 3. Comparing the gap shape characteristics for the three types (Tables 1 and 3), gap type 2 showed the smallest gap size but the largest PARA and FRAC values. The shape of gap type 2 was more elongated than the other two types. The smallest value for CONTIG for gap type 2 indicated that gaps in this category had lesser contiguity or connectedness. The shape characteristics for gap type 1 were similar to those for gap type 2, with a smaller gap size and higher shape complexity. However, larger contiguous gaps resulted in a greater CONTIG value. Finally, the average size of gap type 3 was one order of magnitude greater than the other two gap types. On average, gap type 3 had the smallest values for the fractal dimension but the largest gap size of the three gap types. It was also seen in Fig. 1 that the shape of gap type 3 was more rectangular or square than the other two gap types. The largest CONTIG values for gap type 3 showed the high proximity gap configuration. In conclusion, gap type 1 had a more complex shape, gap type 2 was more elongated and gap type 3 had the largest gaps that are more regular in shape.

### A comparison between gaps types from the two periods

To determine the change in gap types between the two periods, a comparison is shown in Table 4. The largest increase in gap counts was observed for gap type 2, with an increase of 99, from 30 to 129. Consistently, the area of gap type 2 increased greatly from 3.29% (1506 m^2^) to 19.89% (9031 m^2^) in all gap areas. Gap type 1 showed the second largest increase, from 85 to 114. The area of gap type 1 over the entire gap areas increased slightly from 21.61% to 23.97%, which was a minor increase of 2.36%. Gap type 3 accounted for more than half of all of the gap areas in the Lienhuachih Experimental Forest for both periods. The count for gap type 3 declined significantly from 103 to 58 and the area that was occupied by gap type 3 also decreased from 75.10% to 56.14%. The transition matrix of the observed gap type changes based on the gap maps in 1998 and 2002 is shown in Table 5. In the transition matrix, gaps distribution of 1998 and 2002 were indicated in the column total and row total, respectively. Obviously, most small size gaps such as gap type 1 and 2 had recovered during the study period. Only 15.3% of gap type 3 in 1998 was remained canopy opening in 2002. This indicated a high proportion of gaps in 2002 were newborn gaps.

### The association between gap type and forest vegetation type

**Figure 2:**
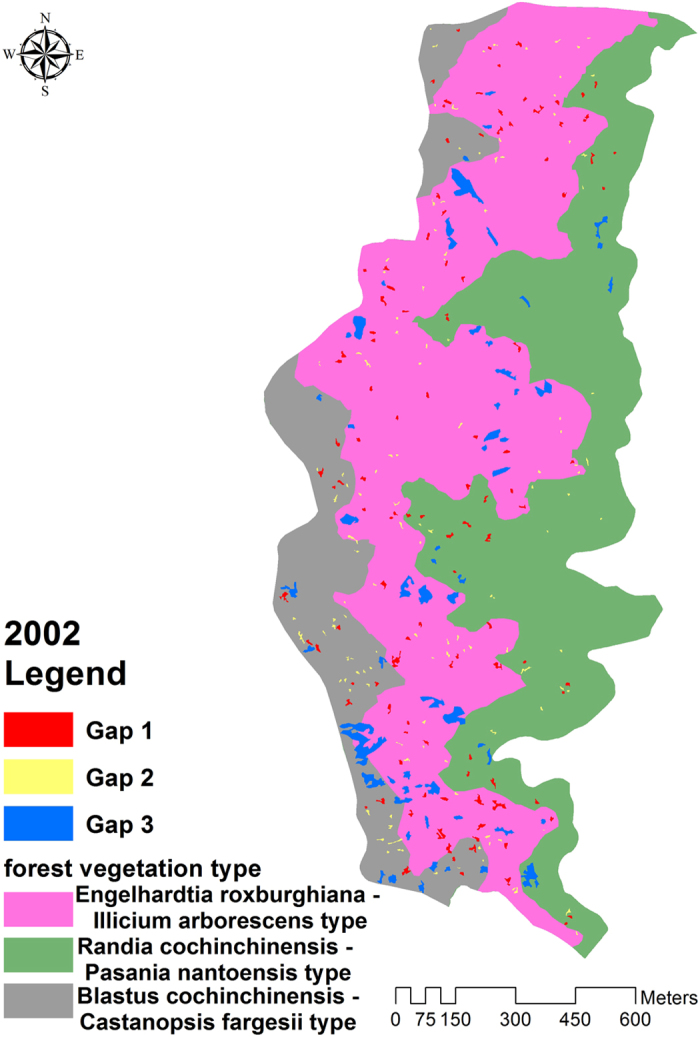
The classified gap distribution in 2002 covered by the three forest vegetation types. Esri ArcGIS 10.2 was used to create this figure (http://www.esri.com/software/arcgis).

Take Lienhuachih Experimental Forest for example, the composition of the forest type varied with terrain or micro-climate in the Lienhuachih Experimental Forest. Figure 2 shows the spatial distribution for each forest vegetation type, overlaid by the filtered three gap types in 2002. *Randia cochinchinensis* - *Pasania nantoensis* type were located around upper slope area. The patch number composition and area of gap types 1, 2, and 3 were 22:23:10 (1:1.05:0.45), and 1905 m^2^, 1369 m^2^, and 2142 m^2^ (0.35%, 0.25%, 0.39%), respectively. *Engelhardtia roxburghiana* - *Illicium arborescens* type was mainly distributed in mid gentle slopes, and the numbers and areas for gap types 1, 2, and 3 were 69:68:35 (1:0.99:0.51), and 6598 m^2^, 2975 m^2^, and 19061 m^2^ (0.90%, 0.41%, 2.61%). *Blastus cochinchinensis* - *Castanopsis fargesii* type was distributed on lower slopes and in valleys with shade and less sunshine. The ratio of counts, and the areas of gap types 1, 2 and 3 was 23:38:13 (1:1.65:0.57), and 2378 m^2^, 4687 m^2^, and 4282 m^2^ (1.04%, 2.04%, 1.87%). The results of chi-squared test showed significant differences in gap count among the three forest types (χ^2^ = 70.79, p = 0.00). This case showed a preliminary attempt to use gap type classification to monitor a forest ecosystem. However, forest structure, growth, and competition were all dynamic and vary with time, so dynamic changes in classified gap types must be included in follow-up research.

## Discussion

This study was the first to propose a shape-based methodology for gap classification, using quantitative landscape indices and multivariate statistics. The results for the Wilks’ Lambda was satisfactory (p < 0.001), with an agreement rate of more than 96%. Differences in gap characteristics between the classified gap types that were analyzed using a one-way ANOVA showed a statistical significance (p = 0.00) in all quantified patch indices, except for ENN for 2002. Taken together, these results demonstrated the robustness and applicability of the proposed gap classification methodology. All the information for this process can be acquired from aerial photographs and GIS platforms so the proposed methodology could be widely applied to other regions and forest landscapes. It is particularly useful to determine the complex functionalities of canopy gaps in forest ecosystem succession, since hundreds or thousands of individual gaps could be simplified into several relevant categories.

Gap classification might give an explanation for the causes of gaps. Using quantified landscape indices and GIS mapping, clear differences in both the shape and areas of gap types can be observed. In this study, it was seen that small gaps, such as gap types 1 and 2, increased in the Lienhuachih Experimental Forest, probably because of canopy breaking, or gully or gap fragmentation, since most of these gaps were small and had a more complete shape, such as rectangular or circular. Several larger canopy openings of gap type 3 were also seen. Landslides or clear cutting were potential reasons for these areas. This information was particularly important for forest ecological studies because different disturbances or causes could denote the succession stages of forest landscapes.

The study area was located in the natural protected environment of Lienhuachih Experimental Forest. Aerial photos and gap data for this area have not yet been made available to the public, so only gap classifications from 1998 and 2002 were used for illustration. However, this does not limit the utility of the proposed gap classification methodology. Lidar scanning technologies are widely used for canopy gap studies because spatial and temporal variations in the distribution of gaps can be accurately assessed using lidar, at relatively low cost. For example, Cifuentes^22^ used a terrestrial laser scanner (TLS) to determine the three-dimensional structure of canopy features. Chena^23^ used airborne waveform lidar to retrieve canopy gap fractions for topography and survey characteristics. Future studies could integrate lidar-based gap data with the proposed gap pattern classification scheme to obtain multi-temporal gap-type dynamics.

In this study, multivariate statistics, including non-hierarchical cluster analysis and canonical discriminant analysis, were used to classify gap types. Several statistical indices were used to determine classification agreement rate, such as Wilks’ Lambda and confusion matrices. The ecological motivation for the classification of gap shape was that gaps with different characteristics may have been created by different processes and may experience different regeneration dynamics. These classification results could be used to determine whether there are ecological differences between the gap classes. Future studies should focus on this aspect if the gap regeneration dynamic data is available.

## Methods

### Study area and material

**Figure 3:**
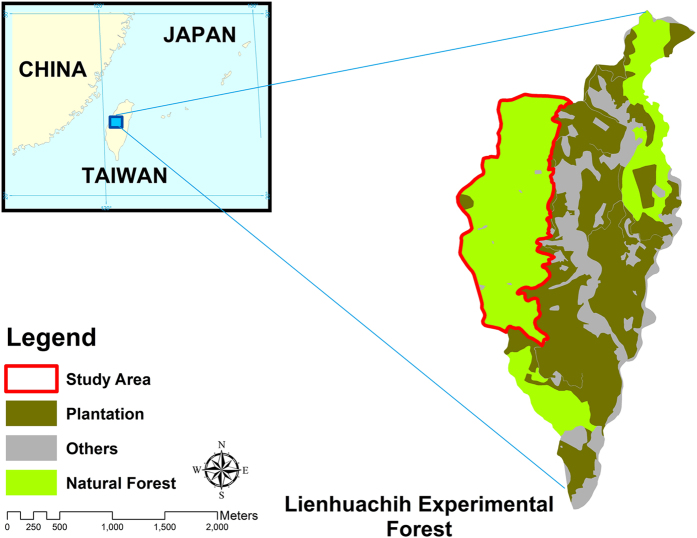
The study area - Lienhuachih Experimental Forest. Esri ArcGIS 10.2 was used to create this figure (http://www.esri.com/software/arcgis).

The study area selected for empirical analysis was Sections 4, 6 and 7 of the Lienhuachih Experimental Forest, which was natural protected forest in central Taiwan (Fig. 3). Managed by the Taiwan Forestry Research Institute (TFRI), the forest has been dedicated to forestry research since 1943 and is in the *Machilus*-*Castanopsis* zone. The total area covers about 150 ha, with elevation ranging from 576 to 925 m. The yearly average temperature is 20.1 °C. The annual prescription is about 2501 mm, with approximately 2241 mm falling during the growing season (April to October). About half of the Lienhuachih Experimental Forest is composed of natural forests and the other half comprises various artificial plantations.

The main vegetation types in the Lienhuachih Experimental Forest include *Randia cochinchinensis* - *Pasania nantoensis* type, *Engelhardtia roxburghiana* - *Illicium arborescens* type and *Blastus cochinchinensis* - *Castanopsis fargesii* type. In total, 879 vascular plant species, from 177 families and 561 genera, have been recorded within the entire Lienhuachih Experimental Forest^24^. Lauraceae (12 species), Rubiaceae (10 species) and Fagaceae (10 species) are the most common species. *Randia cochinchinensis* (Rubiaceae), *Engelhardtia roxburghiana* (Juglandaceae), *Schefflera octophylla* (Araliaceae), *Pasania nantoensis* (Fagaceae), and *Blastus cochinchinensis* (Melastomataceae) are the five dominant species.

**Figure 4:**
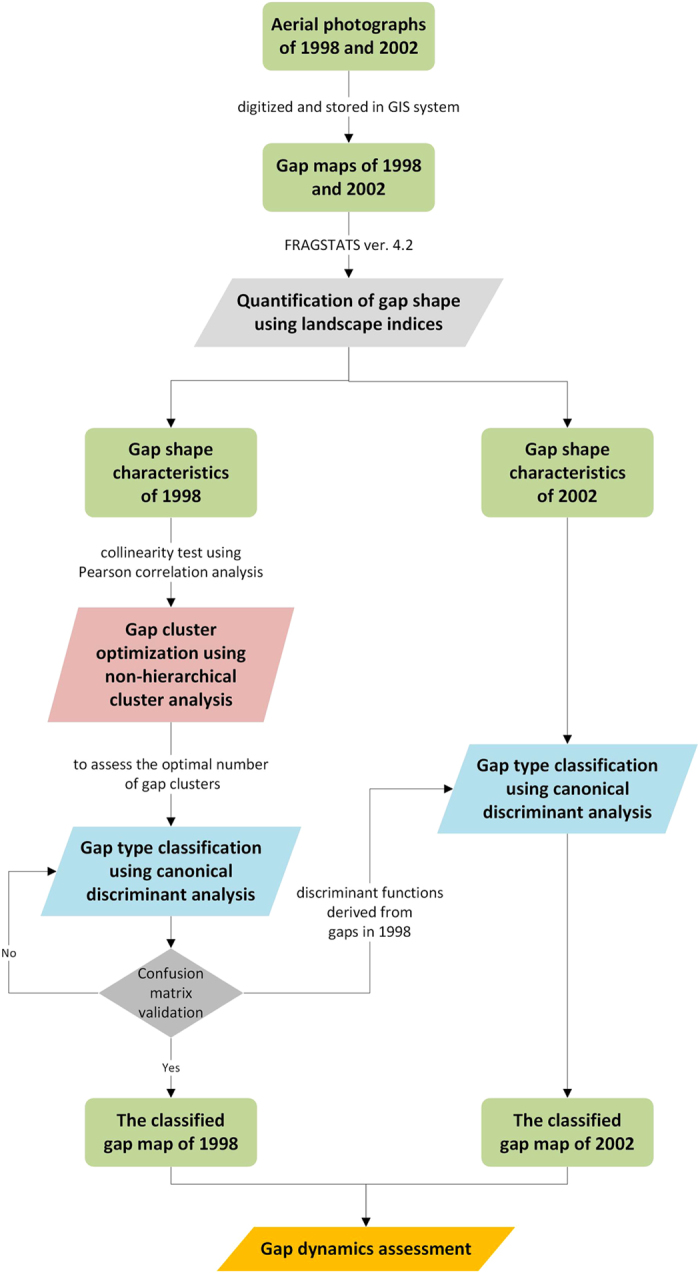
Study framework.

**Figure 5:**
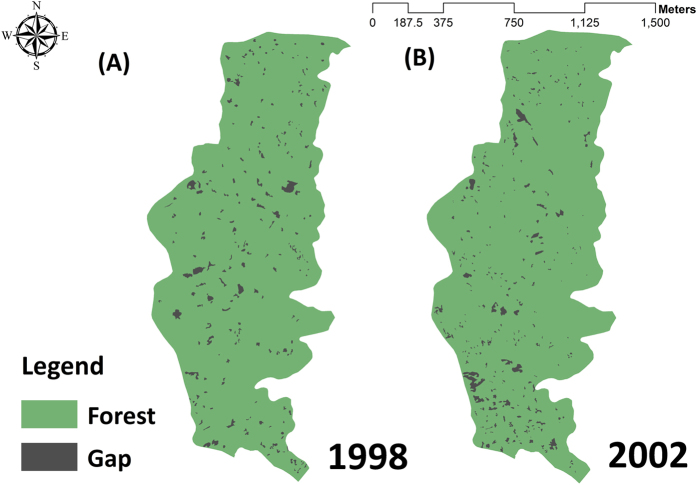
Spatial distribution of gaps in (**A**) 1998 and (**B**) 2002. Esri ArcGIS 10.2 was used to create this figure (http://www.esri.com/software/arcgis).

Figure 4 shows the study framework. Aerial photographs that were taken in 1998 and 2002 were used to delineate the spatial distribution of gaps under a digital stereophotogrammetry workstation. The stereophotogrammetry mapping platform ensures that every single canopy gap was easily detected by seeing through tree crowns in 3D. Images were digitized and stored in the ArcGIS system, with a 1 m × 1 m grid resolution. Figure 5 shows the gap maps for 1998 and 2002. Several programs were used in this study. ArcGIS 10.2 was used for the spatial mapping of canopy gaps. FRAGSTATS version 4.2^19^ was used to calculate the landscape indices to characterize the shape and pattern of gaps. SAS 9.4 and SPSS 22 were respectively used for non-hierarchical cluster and canonical discriminant analysis.

### Quantification of the gap shape using landscape indices

Landscape indices can be defined at three levels that correspond to a logical hierarchical organization of spatial heterogeneity in patch mosaics: patch, class, and landscape levels. Patch metrics are defined for individual patches and characterize the spatial character and context of patches^19^. In this study, each gap was identified as a patch in the forest landscape. Ten patch level indices that were used extensively in previous studies were selected as metrics to quantify the size, shape characteristics and the spatial configuration of gaps, including the patch area (AREA; the area of the gap), the patch perimeter (PERIM; the perimeter of the gap), the radius of gyration (GYRATE; a measure of patch extent, equals the mean distance between each cell in the gap and the gap centroid), the perimeter-area ratio (PARA; the ratio of the gap perimeter to area), the shape index (SHAPE; a measure of the complexity of gap shape compared to a standard shape (square) of the same size), the fractal dimension (FRAC; a measure of the increase in gap shape complexity), the related circumscribing circle (CIRCLE; a measure of gap shape based on ratio of gap area to the area of the smallest circumscribing circle), the contiguity index (CONTIG; a measure of the spatial connectedness, or contiguity, of cells within a grid-cell gap to provide an index on gap boundary configuration and thus gap shape), the proximity index (PROX; the size and distance to all neighboring gaps were enumerated to provide an index of gap isolation.), and the Euclidean nearest neighbor distance (ENN; the distance to the nearest neighboring gap)^25^,^26^,^27^,^28^,^29^,^30^. AREA, PERIM, and GYRATE estimated gap size and edge characteristics; PARA, SHAPE, FRAC, CIRCLE, and CONTIG represented gap shape complexity; PROX and ENN assessed the tendency of gaps to be spatially aggregated or isolated. An unpaired t-test was used to compare the gap shape characteristics between 1998 and 2012. A Pearson correlation matrix was then calculated. Indices with a correlation coefficient greater than 0.6 are excluded, to avoid collinearity during the following clustering procedures.

### Gap cluster optimization using non-hierarchical cluster analysis

In this study, non-hierarchical k-means cluster analysis was firstly used to assess the optimal number of gap clusters for 1998, using the quantified patch indices. Cluster analysis is a statistical approach that categorizes subgroups into groups, according to the similarity and homogeneity of data. The differences within groups are minimized and differences between groups are maximized^31^. The values of the selected indices were standardized, to remove the scale effects. A cubic cluster criterion (CCC) was the optimization criterion for disjointed clusters of gaps. Peaks on the plot with a CCC value greater than 2 or 3 indicated good clusters^32^. A one-way ANOVA confirmed the statistical difference between the classified gap clusters for structural characteristics.

### Gap type classification using canonical discriminant analysis

Canopy gaps for 1998 were classified into the optimal number of categories using canonical discriminant analysis and stepwise variable selection procedures. Canonical discriminant analysis is a dimension-reduction technique that is related to principal component analysis and canonical correlation. Given a nominal classification variable and several interval variables, canonical discriminant analysis derives canonical variables (linear combinations of interval variables) that summarize between-class variation in much the same way that principal components summarize total variation^33^. The standardized canonical discriminant function coefficients compared variables that were measured using different scales. Coefficients with a large absolute value correspond to variables that had a greater discriminatory ability^33^. The model Wilks’ lambda confirmed the performance of the established discriminant functions. Wilks’ lambda is a measure of how well each function separates cases into groups. A smaller value for Wilks’ lambda indicates a greater discriminatory ability for the function. Confusion matrices were produced using self-classification and cross-validation approaches, to assess the percentage of agreement of classification between the use of the developed discriminant functions and the gap classification of 1998 obtained from the cluster analysis. The calculation of agreement rate was expressed as:


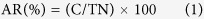


where, AR (%) is the agreement rate, C is the number of gaps that are consistently classified, and TN is the total number of gaps.

The discriminant functions that were derived using the gaps for 1998 were used to classify the gaps for 2002, using the same criterion. A one-way ANOVA determined the differences in gap characteristics between the classified gap types. Finally, a transition matrix was calculated based on the gap maps in 1998 and 2002 to assess the recover trajectories of the classified gap types.

### Assessment of gap type combination for various forest vegetation types

To link gap classification with forest management, the boundaries of three forest vegetation types for the study site for 2002 were digitized on the ArcGIS system, including *Randia cochinchinensis* - *Pasania nantoensis* type, *Engelhardtia roxburghiana* - *Illicium arborescens* type and *Blastus cochinchinensis* - *Castanopsis fargesii* type. Different forest vegetation types were assumed to result in a different gap shape and gap composition. The proportions of the three classified gap categories were calculated within each forest vegetation type. A chi-squared test with a consideration of the different areas of the forest vegetation types was used to evaluate the differences in occurrence of gap types between forest vegetation types. For the detailed calculations of the analysis, please see the Supplementary Materials.

## Additional Information

**How to cite this article**: Wu, C.-D. *et al*. Gap Shape Classification using Landscape Indices and Multivariate Statistics. *Sci. Rep.* **6**, 38217; doi: 10.1038/srep38217 (2016).

**Publisher’s note:** Springer Nature remains neutral with regard to jurisdictional claims in published maps and institutional affiliations.

## Supplementary Material

Supplementary Materials

## Figures and Tables

**Table 1:** Comparison of gap types in 1998 using one-way ANOVA.

Index	Gap type	Number	Average	F statistics	p value
AREA (m^2^)	1	85	111.27	23.65	0.00**
	2	30	50.19		
	3	103	337.32		
	Sum	218	207.30		
PARA	1	85	6436.56	477.02	0.00**
	2	30	9957.61		
	3	103	3880.26		
	Sum	218	5713.32		
FRAC	1	85	1.19	27.78	0.00**
	2	30	1.24		
	3	103	1.14		
	Sum	218	1.17		
CONTIG	1	85	0.82	476.11	0.00**
	2	30	0.72		
	3	103	0.89		
	Sum	218	0.84		
ENN	1	85	3.80	3.42	0.03*
	2	30	1.81		
	3	103	3.77		
	Sum	218	3.51		

^*^Indicates p < 0.05.

^**^Indicates p < 0.01.

**Table 2:** Standardized coefficients of canonical discriminant functions of 1998.

	Function 1	Function 2
AREA	−0.43	1.06
PARA	−0.31	2.17
FRAC	−0.05	−0.51
CONTIG	0.84	1.82
ENN	0.07	0.22

**Table 3:** Comparison of gap types in 2002 using one-way ANOVA.

Index	Gap type	Number	Average	F statistics	p value
AREA (m^2^)	1	114	95.45	236.74	0.00**
	2	129	70.01		
	3	58	439.40		
	Sum	301	141.44		
PARA	1	114	6482	133.64	0.00**
	2	129	11449.36		
	3	58	1941.18		
	Sum	301	7950.91		
FRAC	1	114	1.16	120.67	0.00**
	2	129	1.28		
	3	58	1.18		
	Sum	301	1.20		
CONTIG	1	114	0.81	269.53	0.00**
	2	129	0.69		
	3	58	0.89		
	Sum	301	0.78		
ENN	1	114	20.97	0.192	0.83
	2	129	19.60		
	3	58	19.96		
	Sum	301	20.19		

*Indicates p < 0.05.

**Indicates p < 0.01.

**Table 4:** Comparison of gap types between 1998 and 2002.

Gap type	1998		2002		Difference^b^	
	Number	Area (m^2^)	Number	Area (m^2^)	Number	Area (m^2^)
Gap type 1	85	9903 (21.61%)^a^	114	10881 (23.97%)	29	978 (2.36%)
Gap type 2	30	1506 (3.29%)	129	9031 (19.89%)	99	7525 (16.60%)
Gap type 3	103	34407 (75.10%)	58	25485 (56.14%)	−45	−8922 (−18.96%)
Total	218	45816 (100%)	301	45397 (100%)	83	−419

^a^The number in parentheses denotes the gap percentage over the entire gap area.

^b^2002–1998.

**Table 5:** Transitional areas (m^2^) and probabilities from 1998 to 2002.

Transition from column to row	Forest	Gap type 1	Gap type 2	Gap type 3	Row total (2002 distribution)
Forest	1418700 (0.974)^a^	9217 (0.931)	1422 (0.944)	28314 (0.823)	1457653
Gap type 1	9199 (0.006)	385 (0.039)	46 (0.031)	1251 (0.036)	10881
Gap type 2	7914 (0.005)	132 (0.013)	38 (0.025)	947 (0.028)	9031
Gap type 3	21421 (0.015)	169 (0.017)	0	3895 (0.113)	25485
Column total (1998 distribution)	1457234	9903	1506	34407	1503050

In the transition matrix, gaps distribution of 1998 and 2002 were indicated in the column total and row total.

^a^The number in parentheses denotes the probability.
