# Supplementary material for: Gap Shape Classification using Landscape Indices and Multivariate Statistics
Source: Sci Rep. 2016 Nov 30;6:38217. doi: 10.1038/srep38217 (PMC5128799; doi:10.1038/srep38217)
Supplement: Supplementary Materials [file srep38217-s1.doc]

**Gap Shape Classification using Landscape Indices and Multivariate Statistics**

**Chih-Da Wu^1^, Chi-Chuan Cheng^2^, Che-Chang Chang^3^, Chinsu Lin^1^,**

**Kun-Cheng Chang^1^, and Yung-Chung Chuang^4,*^**

**^1^ Department of Forestry and Natural Resources, College of Agriculture, National Chiayi University, Chiayi, 60004, Taiwan.**

**^2^ The Department and Graduate Institute of Landscape Architecture, Chinese Culture University, Taipei, 11114, Taiwan.**

**^3^ Technical Service Division, Taiwan Forestry Research Institute, Taipei, 10066, Taiwan.**

**^4^ Department of the Urban Planning and Spatial Information, Feng Chia University, Taichung, 407, Taiwan.**

**^*^ yungcchuang@fcu.edu.tw**

**Supplementary Materials**

**Table S1**. Gap statistics in 1998 and 2002

|  | 1998 | | 2002 | |
| --- | --- | --- | --- | --- |
|  | Gap | Forest | Gap | Forest |
| Number | 218 | 1 | 301 | 1 |
| Area (m^2^) | 45816 (3.04%) | 1457234 (96.96%) | 45397 (3.02%) | 1457653 (96.98%) |
| Total Area (m^2^) | 1503050 (100%) | | 1503050 (100%) | |

* The number in parenthesis denotes the gap percentage over the entire study area.

**Table S2.** Un-paired t-test of gap characteristics between the two years

| Index | Year | Mean | Standard Deviation | t statistic | p value |
| --- | --- | --- | --- | --- | --- |
| AREA  (m^2^) | 1998 | 207.30 | 299.05 | 2.00 | 0.045 |
|  | 2002 | 141.44 | 339.24 |  |  |
| PARA | 1998 | 5713.32 | 2292.82 | -8.57 | 0.00** |
|  | 2002 | 7943.89 | 3622.79 |  |  |
| FRAC | 1998 | 1.17 | 0.08 | -3.75 | 0.00** |
|  | 2002 | 1.20 | 0.09 |  |  |
| CONTIG | 1998 | 0.84 | 0.06 | 8.58 | 0.00** |
|  | 2002 | 0.78 | 0.10 |  |  |
| PROX | 1998 | 3.51 | 8.65 | 1.67 | 0.10 |
|  | 2002 | 2.43 | 4.61 |  |  |
| ENN | 1998 | 24.75 | 19.43 | 2.80 | 0.01 |
|  | 2002 | 20.19 | 17.44 |  |  |

* indicates p < 0.05

** indicates p < 0.01

**Table S3.** Correlation coefficients among the ten patch indices in 1998

|  | AREA | PERIM | GYRATE | PARA | SHAPE | FRAC | CIRCLE | CONTIG | PROX | ENN |
| --- | --- | --- | --- | --- | --- | --- | --- | --- | --- | --- |
| AREA | 1.00 |  |  |  |  |  |  |  |  |  |
| PERIM | 0.85 | 1.00 |  |  |  |  |  |  |  |  |
| GYRATE | 0.83 | 0.98 | 1.00 |  |  |  |  |  |  |  |
| PARA | -0.42 | -0.45 | -0.48 | 1.00 |  |  |  |  |  |  |
| SHAPE | 0.22 | 0.61 | 0.60 | -0.01 | 1.00 |  |  |  |  |  |
| FRAC | -0.05 | 0.29 | 0.27 | 0.21 | 0.90 | 1.00 |  |  |  |  |
| CIRCLE | 0.03 | 0.32 | 0.36 | -0.01 | 0.75 | 0.80 | 1.00 |  |  |  |
| CONTIG | 0.45 | 0.48 | 0.51 | -1.00 | 0.01 | -0.23 | -0.01 | 1.00 |  |  |
| PROX | 0.14 | 0.11 | 0.09 | 0.14 | 0.00 | -0.04 | -0.03 | -0.12 | 1.00 |  |
| ENN | 0.00 | 0.02 | 0.04 | -0.16 | -0.04 | -0.09 | -0.01 | 0.16 | -0.39 | 1.00 |

**Table S4.** Model Wilks' Lambda of 1998

| Test of functions | Wilks' Lambda | Chi-square | p value |
| --- | --- | --- | --- |
| 1 | 0.15 | 404.25 | 0.00** |
| 2 | 0.94 | 14.32 | 0.006** |

** indicates p < 0.01

**Table S5.** Confusion matrices of gap classification of 1998a, b

| Approaches | Type | 1 | 2 | 3 | Total |
| --- | --- | --- | --- | --- | --- |
| Self-classification | 1 | 83 | 0 | 2 | 85 |
|  | 2 | 0 | 30 | 0 | 30 |
|  | 3 | 4 | 0 | 99 | 103 |
| Cross-validation | 1 | 82 | 0 | 3 | 85 |
|  | 2 | 0 | 30 | 0 | 30 |
|  | 3 | 5 | 0 | 98 | 103 |

^a^ 97.2% of original grouped cases consistently classified.

^b^ 96.3% of cross-validated grouped cases consistently classified.

**The calculations of the chi-squared test considering the different areas of the forest vegetation types**

A chi-squared test with a consideration of the different areas of the forest vegetation types was used to evaluate the differences in occurrence of gap types between forest vegetation types. In this case, the expected values were estimated using a priori expectations, rather than empirically from the row and column totals in the contingency table. The forest vegetation type totals were adjusted in the chi-squared contingency table to match the number of gaps that would be expected if all gaps were distributed proportionate to the area of the forest types (eg, 36% of all gaps to type R-P, 49% to E-I, 15% to B-C), and use those estimates to calculate the expected numbers of gaps and perform the chi-squared test. To show the details:

**THE OBSERVED VALUES**

|  | **R-P** | **E-I** | **B-C** | **Row_TOTAL** |
| --- | --- | --- | --- | --- |
| **Gap 1** | 22 | 69 | 23 | 144 |
| **Gap 2** | 23 | 68 | 38 | 129 |
| **Gap 3** | 10 | 35 | 13 | 58 |
| **Col_TOTAL** | 55 | 172 | 74 | 301 |

EXPECTED VALUES CALCULATED USING A PRIORI COLUMN TOTALS FOR FOREST TYPE AND OBSERVED ROW TOTALS FOR GAP TYPE

|  | **R-P** | **E-I** | **B-C** | **Row_TOTAL** |
| --- | --- | --- | --- | --- |
| **Gap 1** | 41 | 55 | 57 | 144 |
| **Gap 2** | 47 | 63 | 20 | 129 |
| **Gap 3** | 21 | 28 | 9 | 58 |
| **Col_TOTAL** | 109 | 146 | 46 | 301 |

**((**OBSERVED VALUES – EXPECTED VALUES)^2)/EXPECTED VALUES

|  | **R-P** | **E-I** | **B-C** |
| --- | --- | --- | --- |
| **Gap 1** | 8.80 | 3.56 | 20.28 |
| **Gap 2** | 12.26 | 0.40 | 16.20 |
| **Gap 3** | 5.76 | 1.75 | 1.78 |

χ^2^= 8.8 + 12.26 + 5.76 + 3.56 + 0.4 + 1.75 + 20.28 +16.2 +1.78 = 70.79,

> ref-χ^2^ (0.05, 4) = 9.49, indicates the statistically significant differences in gap count among the three forest types.
